# Elucidation of How Cancer Cells Avoid Acidosis through Comparative Transcriptomic Data Analysis

**DOI:** 10.1371/journal.pone.0071177

**Published:** 2013-08-14

**Authors:** Kun Xu, Xizeng Mao, Minesh Mehta, Juan Cui, Chi Zhang, Fenglou Mao, Ying Xu

**Affiliations:** 1 Computational Systems Biology Lab, University of Georgia, Athens, Georgia, United States of America; 2 Department of Biochemistry and Molecular Biology and Institute of Bioinformatics, University of Georgia, Athens, Georgia, United States of America; 3 Department of Statistics, University of Georgia, Athens, Georgia, United States of America; 4 Department of Internal Medicine, University of Cincinnati, Cincinnati, Ohio, United States of America; 5 College of Computer Science and Technology, Jilin University, Changchun, Jilin, China; Queen’s University Belfast, United Kingdom

## Abstract

The rapid growth of cancer cells fueled by glycolysis produces large amounts of protons in cancer cells, which tri mechanisms to transport them out, hence leading to increased acidity in their extracellular environments. It has been well established that the increased acidity will induce cell death of normal cells but not cancer cells. The main question we address here is: how cancer cells deal with the increased acidity to avoid the activation of apoptosis. We have carried out a comparative analysis of transcriptomic data of six solid cancer types, breast, colon, liver, two lung (adenocarcinoma, squamous cell carcinoma) and prostate cancers, and proposed a model of how cancer cells utilize a few mechanisms to keep the protons outside of the cells. The model consists of a number of previously, well or partially, studied mechanisms for transporting out the excess protons, such as through the monocarboxylate transporters, V-ATPases, NHEs and the one facilitated by carbonic anhydrases. In addition we propose a new mechanism that neutralizes protons through the conversion of glutamate to γ-aminobutyrate, which consumes one proton per reaction. We hypothesize that these processes are regulated by cancer related conditions such as hypoxia and growth factors and by the pH levels, making these encoded processes not available to normal cells under acidic conditions.

## Introduction

One of the key cancer hallmarks is their reprogrammed energy metabolism [1]. That is, glycolysis replaces oxidative phosphorylation to become the main ATP producer. A direct result of this change is that substantially more lactates, as the terminal receivers of electrons from the glucose metabolism, are produced and transported out of the cells. To maintain the cellular electro-neutrality when releasing lactates, the cells release one proton for each released lactate, the anionic form of lactic acid. This leads to increased acidity in the extracellular environment of the cancer cells. It has been well established that high (extracellular) acidity can induce the apoptotic process in normal cells [2], leading to their death. Interestingly this does not seem to happen to cancer cells, hence giving them a competitive advantage over the normal cells and allowing them to encroach the space occupied by the normal cells. Currently it is not well understood of how the cancer cells deal with the increased acidity in their extracellular environments to avoid acidosis.

A number of studies have been published focused on issues related to how cancer cells deal with the increased acidity in both the extracellular and intracellular environments [3], [4], [5], [6], [7], [8], [9]. The majority of these studies were focused on possible cellular mechanisms for transporting out or neutralizing intracellular protons, typically focused on one cancer type. More importantly these studies did not tie such observed capabilities and proposed mechanisms of cancer cells in avoiding acidosis with the rapid growth of cancer as we suspect there is an encoded mechanism that connects the two.

We have carried out a comparative analysis of genome-scale transcriptomic data on six types of solid cancers, namely breast, colon, liver, two lung (adenocarcinoma, squamous cell carcinoma) and prostate cancers, aiming to gain a systems level understanding of how the cancer cells keep their intracellular pH level within the normal range while their extracellular pH level is low. Our analysis, focused on transporters and enzymes, of the transcriptomic data on these cancer and their matching control tissues indicate that (i) all the six cancer types utilize the monocarboxylate transporters as the main mechanism to transport out lactates and protons simultaneously, triggered by the accumulation of intracellular lactates; (ii) these transporters are probably supplemented by additional mechanisms through anti-porters such as ATPases to transport protons out in exchange of certain cations such as Ca^2+^ or Na^+^ to reduce the intracellular acidity while maintaining the cellular electron-neutrality; and (iii) cancer cells may also utilize another mechanism, i.e., using glutamate decarboxylase to catalyze the decarboxylation of glutamate to a γ-aminobutyric acid (GABA), consuming one proton for each reaction – a similar process is used by the bacterial *Lactococcus lactis* to neutralize acidity when lactates are produced. Based on these analysis results, we proposed a model that connects these deacidification processes with a number of cancer related genes/cellular conditions, which are probably intrinsic capabilities of fast-growing cells used under hypoxic conditions rather than gained capabilities through molecular mutations.

We believe that our study represents the first systemic study focused on how cancer cells deal with the acidic environment through the activation of the encoded acid resistance mechanisms triggered by cancer associated genes and conditions. These results have established a foundation for a novel model for how cancer cells avoid acidosis.

## Results

### 1. Cellular Responses to Increased Acidity

The degradation of each mole of glucose generates 2 lactates, 2 protons and 2 ATPs, detailed as
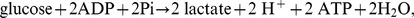
showing the source of the increased acidity when glycolysis serves as the main ATP producer in cancer cells [10]; in contrast the complete degradation of glucose through oxidative phosphorylation is pH neutral. Clearly these extra protons need to be removed or neutralized since otherwise they will induce apoptosis. The monocarboxylate transporter (MCT), specifically the SLC16A family, has been reported to play a key role in maintaining the pH homeostasis [11] with four isoforms, MCT1 - 4, playing crucial roles in proton-linked transportation [12], [13]. Previous studies have reported that the MCT1, MCT2 and MCT4 genes are up-regulated in cancer such as in breast, colon, lung and ovary cancers [14], [15]. It has also been observed that a monocarboxylate transporter pumps out lactates and protons with a 1∶1 stoichiometry to maintain cellular electron-neutrality [16].

**Figure 1:**
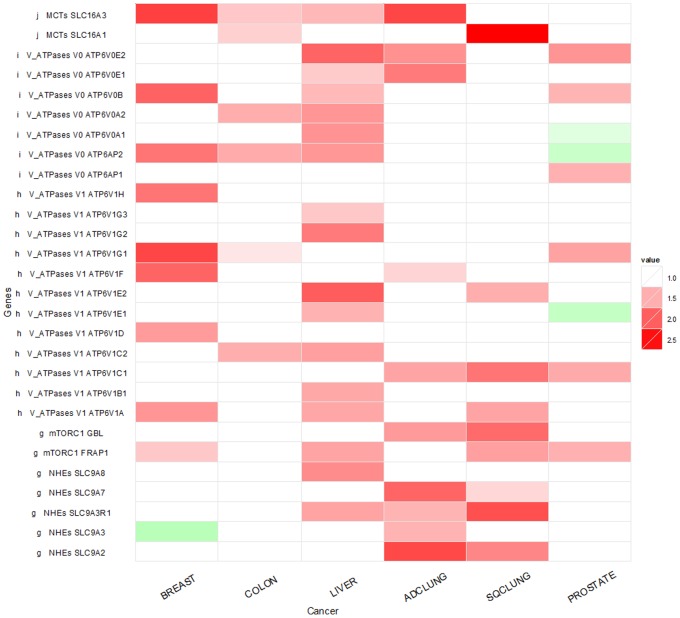
Expression-level changes of V-ATPase and related genes in six cancer types in comparison with their matching control tissues. Each entry in the table shows the ratio between a gene’s expression levels in cancer and the matching control, averaged across all the samples.

Our transcriptomic data analyses of the six cancer types added to this knowledge that these MCT genes also show up-regulation in five out of the six cancer types. The only exception is the prostate cancer, which did not show any increased expression of the MCT genes. Figure 1 shows the transcription up-regulation of MCT1 (SLC16A1) and MCT4 (SLC16A3) in five cancer types. Specifically MCT4 shows up-regulation in four of the six cancer types, an observation that has not been reported before.

One published study suggests that MCT1 might be regulated by p53 [17] in cancer. Another study shows strong evidence that MCT1 and MCT4 are regulated by the intracellular level of hypoxia. We hypothesize that hypoxia may be the main regulating factor of the over-expression of the MCT genes, which may require additional conditions such as the pH level or the accumulation of lactates as the co-regulating factors, as suggested by our analysis result of transcriptomic data of cell lines collected under hypoxic condition, where MCT1 and MCT4 genes are up-regulated (see Figure 1 and Figure S1 for details).

The protons transported out of the cells will increase the acidity of the extracellular environment. Previous studies have shown that (normal) cells tend to adjust their intracellular pH level to a similar pH level of the extracellular environment [18]. It has been well established that the increased intracellular acidity will induce apoptosis through directly activating the caspase genes, which bypasses the more upstream regulatory proteins of the apoptosis system such as p53, hence leading to death of the normal cells that do not seem to have the right intracellular conditions to deal with the reduced pH.

### 2. Additional Mechanisms for Dealing with Excess Protons in Cancer Cells

We have examined if other genes may be relevant to the removal or neutralization of protons in cancer cells in a systematic manner across all the human genes. Our main findings are summarized in Figure 1, detailed as follows.

#### V-ATPase

Transmembrane ATPases import many of the metabolites necessary for cellular metabolisms and export toxins, wastes and solutes that can hinder the health of the cells [19]. One particular type of ATPase is the V-ATPase that transports solutes out using ATP hydrolysis as the energy. It pumps out a proton in exchange for an extracellular Na^+^ or another cation such as K^+^ or Ca^2+^ to maintain the intracellular electro-neutrality. V-ATPases have been found to be up-regulated in multiple cancer types, but the previous studies have been mostly focused on using the increased V-ATPase gene expression levels as a biomarker for metastasis [20] or on utilizing them as potential drug targets as a way to trigger apoptosis, hence causing cancer cell death [20], [21], [22].

We have examined the expression levels of the 19 genes that encode the subunits of V-ATPase, the V_0_ (transmembrane) domain and the V_1_ (cytoplasmic) domain, namely ATP6V0A1, ATP6V0A2, ATP6V0B, ATP6V0E1, ATP6V0E2, ATP6AP1 and ATP6AP2 for V_0_ and ATP6V1A, ATP6V1B1, ATP6V1C1, ATP6V1C2, ATP6V1D, ATP6V1E1, ATP6V1E2, ATP6V1F, ATP6V1G1, ATP6V1G2, ATP6V1G3 and ATP6V1H for V_1_. We found that multiple V-ATPase genes are up-regulated, indicating that the V-ATPases are active in transporting the protons out. Interestingly some of the ATPase genes do not show up-regulation and some even show down-regulation in prostate cancer (Figure 1). More detailed examination of the gene expression data indicates that the actual expression levels of the ATPase genes are at the baseline level in both the prostate cancer and the adjacent control issues, hence the fold-change data are not particularly informative. Overall the data on prostate cancer seem to suggest that the acidity level in this cancer type is not substantially elevated. For the other five cancer types, the expression levels of some V-ATPase genes do not show changes in cancer. We note that these gene expression levels are also elevated in the control tissues compared to cell-line data of the matching tissue types (data not shown here), which is consistent with previously published data, suggesting that the elevated acidic level in the extracellular environment can also induce increased expression of the V-ATPase genes in normal tissues [23]. This may explain why some of the V-ATPase genes do not show overexpression in cancer *versus* adjacent control tissues.

Then the question is why cancer cells seem to handle the increased acidity better than the normal cells. Our hypothesis is that while pH may play some regulatory role of the expression of the V-ATPase genes, the main regulator of the V-ATPase is probably mTORC1 as it has been recently suggested [24]. mTORC is one of the most important regulators relevant to cell growth, and it generally has dysregulated expressions in cancer. To check on this hypothesis, we have examined the gene expression level of mTORC1 (the GBL and FRAP1 genes) in the six cancer types. We see clear up-regulation of these genes in all six cancer types as shown in Figure 1. So overall we speculate that it is the combined effect of decreased pH and up-regulation of mTORC1 that makes cancer cells more effective in pumping out the excess protons than the normal cells.

#### Na+-H+ Exchanger (NHE)

NHE anti-porters represent another class of proteins that can transport out protons and exchange for a cation to maintain intracellular electro-neutrality. We have examined the five genes encoding this class of transporters, and found that these genes are highly up-regulated in the two lung cancer types. Interestingly the expression-change patterns are highly complementary between NHE genes and the V-ATPase genes in five out of the six cancer types, specifically up-regulation in breast, colon and liver cancers but not in the two lung cancer types as shown in Figure 1. Hence we speculate that the NHE anti-porters may play a complementary role to that of the V-ATPases through coordinated regulation by an unknown mechanism. Literature search suggests that NHEs are regulated by both growth factors and pH among a few other factors [25], which partially explains why the system is more active in cancer (affected by both growth factors and pH) than in control tissues (affected by pH only).

### 3. Carbonic Anhydrases Play Roles in pH Neutralization in Cancer Cells

It has been previously suggested that carbonic anhydrases (CAs) play a role in neutralizing the protons in cancer cells. For example, a model of how the membrane-associated CAs facilitate out-transportation of protons has been presented [26]. The key idea of the model is that the membrane bound CAs catalyze the otherwise slow reaction from CO_2_+ H_2_O to H_2_CO_3_, which dissociates into HCO_3_^−^ and H^+^ in an acidic extracellular environment, as detailed by
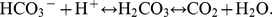



The HCO_3_^−^ (bicarbonate**)** is then transported across the membrane through an NBC transporter [27] into the intracellular environment, where it reacts with a H^+^ to form a CO_2_ and H_2_O; and the CO_2_ is freely membrane-permeable to get outside the cell, forming a cycle for removing some of the excess H^+^. See Figure S1 for a more detailed picture of this mechanism.

**Figure 2:**
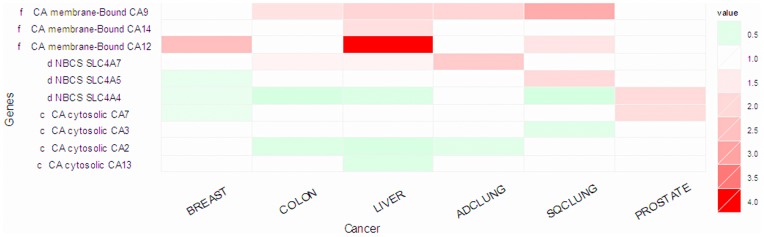
Expression level changes of genes involved in carbonic anhydrases (CAs) pH regulation in six cancer tissues in comparison with their matching control tissues.

To check if the model is supported by the transcriptomic data being analyzed in our study, we note that (1) three membrane-associated CAs (CA9, CA12, CA14) show up-regulation in five out of six cancer types (except for prostate cancer), as shown Figure 2; and (2) two of the three NBC genes, NBC2 (SLC4A5) and NBC3 (SLC4A7), show up-regulation in four cancer types. It has been reported that CA9 and CA12 are hypoxia-inducible in brain cancer [28]. Hence we hypothesize that all the three above membrane-associated CAs are inducible by hypoxia. In addition, our literature search indicates that the NBC genes are pH inducible [29].

Interestingly all the cytosolic CAs (CA2, CA3, CA7, CA13) show down-regulation, reflecting that oxidative phosphorylation is not being used as actively and hence produces less CO_2_ in cancer cells as in normal cells.

### 4. Neutralization of Acidity through Decarboxylation Reactions: A Novel Mechanism?

Our search for possible mechanisms of cancer cells in deacidification led us to study how *Lactococcus lactis* deals with the lactic acids. We note that the bacteria use the glutamate decarboxylases (GAD) to consume one (dissociable) H^+^ during the decarboxylation reaction that it catalyzes [30], as shown below:
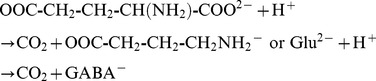



The reaction converts a glutamate to one γ-aminobutyrate (GABA) plus a CO_2_. Two human homologues of the GAD, GAD1 and GAD2, have been found. Published studies have shown that the activation of the GAD genes leads to GABA synthesis in human brain [31], suggesting that the human GAD genes have the same function as the bacterial GAB gene, i.e., catalyzing the reaction for the synthesis of GABA. The majority of these studies were done in the context of the nervous system in human brains [32], [33], [34]. Specifically, GABA is known to serve as a key inhibitory neurotransmitter. In addition, activities of GABA have been found in human liver [35]. While hypotheses have been postulated about its functions in liver [36], no solid evidence has been established about its function there.

**Figure 3:**
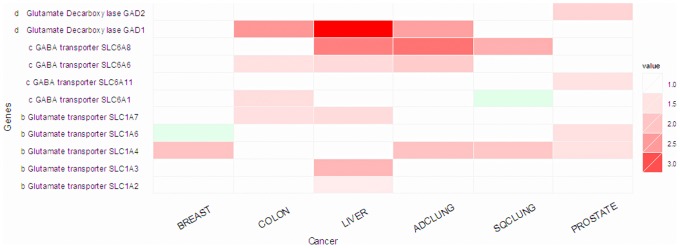
Expression level changes of genes involved in the conversion of glutamate to GABA and CO_2_, along with the genes encoding the GABA transporters.

We have observed that GAD1 is up-regulated in three out six cancer types under study, namely colon, liver and lung adenocarcinoma, and GAD2 is up-regulated in prostate cancer. It has been fairly well established that glutamate, the substrate of the above reaction catalyzed by GAD, is elevated in cancer in general [37]. Hence it makes sense to assume that the above reaction indeed takes place in cancer. This is supported by our observation that multiple in-take transporters of glutamate are up-regulated in five out six cancer types (see Figure 3). An even more interesting observation is that multiple genes encoding the out-going transporters of GABA are up-regulated in five out of the six cancer types, indicating that the GABA molecules are not being used by cancer cells but instead serve a way to remove H^+^ out of the cells.

Currently, to the best of our knowledge no published data are available to implicate which genes encode the main regulator of the GAD genes. Interestingly, our search for possible regulators of the GAD genes in the Cscan database [38] revealed that FOS, a known oncogene, can potentially regulate the GAD genes [39]. Some experimental data from the ENCODE database [40] show that the expression of the GAD1 gene (NM_000817, NM_013445) is positively co-related with that of FOS in the HUVEC cell-line. Integrating this information, we hypothesize that FOS, in conjunction with some pH–associated regulator, regulates the GAD genes, which leads to the synthesis of GABA and reduces one H^+^ as a by-product per synthesized GABA; then the unneeded GABA molecules are transported out of the cells. This may provide another mechanism that cancer cells use to keep their intracellular pH level in the normal range.

### 5. A Model for Cancer Cells to Keep their Intracellular pH in the Normal Range

**Figure 4:**
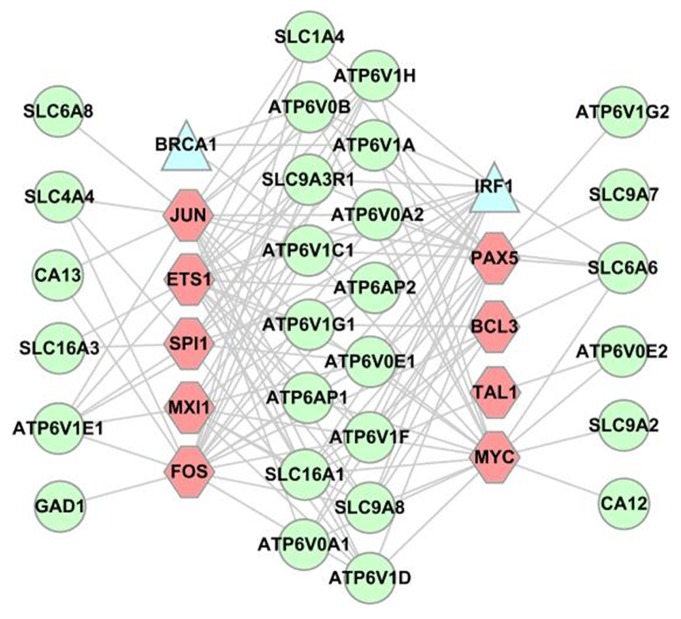
Regulatory relationships between genes involved in deacidification and cancer growth. Each circle represents a deacidification-related gene, each hexgon represents an oncogene and each triangle a tumor suppressor gene, with each link representing a direct regulatory relationship.

Overall 44 genes are implicated in our above analyses. Our search results of these genes against Cscan database [38] indicate that 28 of these genes are regulated directly by nine proto-oncogenes, namely BCL3, ETS1, FOS, JUN, MXI1, MYC, PAX5, SPI1 and TAL1; and 17 genes are regulated by two tumor-suppressors, IRF1 and BRCA1 as shown in Figure 4, indicating that there is a strong connection between deacidification and cancer growth.

**Figure 5:**
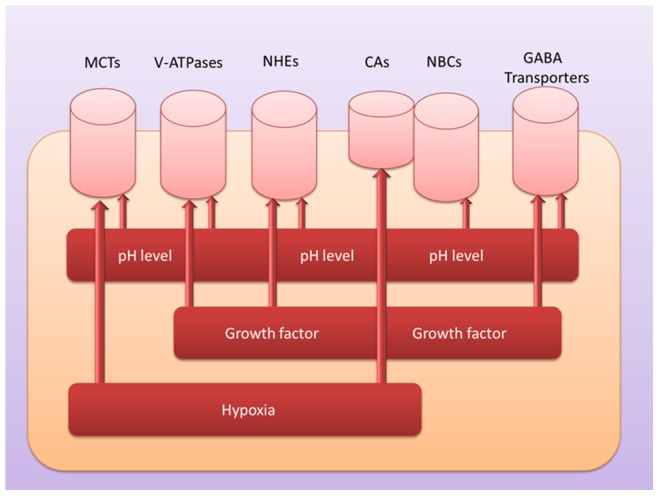
A model for deacdification in cancer cells. Each cylinder represents a pump or transporter used to remove protons and possibly other molecules out of the cell; and each rectangle bar represents a condition that is a possible regulatory factor for the corresponding pump or transporter.

Figure 5 summarizes our overall model for the cellular deacidification mechanisms and the associated conditions that may trigger each mechanism to be activated. Specifically, we hypothesize that hypoxia and growth factors may serve as the main regulatory factors of the deacdification processes, hence making them available only in cancer cells, in conjunction with the cellular pH level.

## Discussion

Based on comparative transcriptomic data analysis results on six cancer types, we have proposed a model of how cancer cells deal with excess protons in both intracellular and extracellular environments, which are generated due to the reprogrammed energy metabolism. Some of the mechanisms have been reported in the literature but mostly in a fewer cancer types. Our analysis results have confirmed and expanded the models previously proposed. In addition we have proposed a new model based on how bacterial *Lactococcus* deals with a similar situation. Another contribution of the work is that we have proposed possible regulatory mechanisms that allow cancer cells to fully utilize these encoded deacidification mechanisms that are not triggered in normal cells.

Since our proposed model is based on transcriptomic data only, further experimental validation on a number of the hypotheses are clearly needed, including (i) the main regulators of these processes and their regulatory relationships with pH related regulators, (ii) the new mechanism proposed based on a homologous system in *Lactococcus*, the organism that produces lactate; and (iii) the proposed NBC cotransporter transports in HCO_3_^−^ and Na^+^ together, but it is not clear how the Na^+^ is handled in cancer cells; and similar questions can be asked about the inported Ca^2+^ or Na^+^ by other deacidification processes. All these require further investigation both experimentally and computationally.

Our overall search procedure for enzymes and transporters that may change the number of protons in a systematic manner proves to be highly effective. For example, the carbonic anhydrases are found to be possibly relevant to the deacidification process from the search; only later we found that this system has been studied and reported in the literature. This result clearly shows the power of this procedure, when coupled with additional searches and analyses of the transcriptomic data, which we believe to be applicable to elucidation of other cancer-related processes.

## Materials and Methods

### 1. Gene Expression Data for Six Cancer Types

The gene-expression data for the six cancer types, (breast, colon, liver, lung adenocarcinoma, squamous cell lung, prostate), are downloaded from the GEO database [41] of the NCBI. For each cancer type, we have applied the following criteria in selecting the dataset used for this study: (1) all the data in each dataset were generated using the same platform by the same research group; (2) each dataset consists of only paired samples, i.e., cancer tissue sample and the matching adjacent noncancerous tissue sample; and (3) each dataset has at least 10 pairs of samples. In the GEO database, only six cancer types have datasets satisfying these criteria. A summary of the 12 datasets, 2 sets for each cancer, is listed in Table 1.

**Table 1:** A summary of the cancer datasets used in our transcriptomic data analysis.

	set1	set2	pairs
breast cancer	GSE14999 [44]	GSE15852 [45]	61/43
colon cancer	GSE18105 [46]	GSE25070 [47]	17/26
liver cancer	GSE22058 [48]	GSE25097 [49]	97/238
lung adenocarcinoma	GSE31552 [50]	GSE7670 [51]	31/26
lung squamous cell carcinoma	GSE31446 [52]	GSE31552 [50]	13/17
prostate cancer	GSE21034 [53]	GSE6608 [54]	29/58

### 2. Identification of Differentially Expressed Genes in Cancer *versus* Control Tissues

For each dataset used in this study, we have used the normalized expression data from the original study. Since we used only paired data, a sign test developed by Wilcoxon [42] for matched pairs, is applied to identify the significant differentially expressed genes in cancer *versus* adjacent normal samples for each dataset. We consider a gene being differentially expressed if the statistical significance, *p*-value, is less than 0.01. For each cancer type, we consider only genes with consistent up- or down-regulation across all samples as differentially expressed genes. The final fold change is calculated by taking the mean of the fold change between the cancer and control samples.

### 3. Searching for Regulatory Relationships in Human

To retrieve the transcriptional regulation relationship information about the genes we are interested in this study, we have used a public database along with its search engine Cscan (http://www.beaconlab.it/cscan) to predict the common transcription regulators based on a large collection of ChIP-Seq data for several TFs and other factors related to transcription regulation for human and mouse [38]. The regulatory relationships were inferred based on ChIP-Seq data collected under 777 different conditions in the hmChip database [43] and transcription factors from the UCSC Genome Browser [40].

### 4. Cancer Related Genes

To retrieve cancer related genes, specifically proto-oncogene and tumor suppressor genes for our study, we searched the UNIPROT database (http://www.uniprot.org/keywords/) using keywords, which led to the retrieval of 232 proto-oncogenes (KW-0656) and 194 tumor-suppressor genes (KW-0043) in human.

## Supporting Information

Figure S1 Deacidification mechanisms in cancer cells. Each rectangle bar represents a transporter, enzyme or pump family. The red colored rectangles are up-regulated in our study and the green show down-regulation. Dashed arrows indicate CO_2_ diffusion across the membrane.
(PDF)
