# Supplementary material for: Elucidation of How Cancer Cells Avoid Acidosis through Comparative Transcriptomic Data Analysis
Source: PLoS One. 2013 Aug 14;8(8):e71177. doi: 10.1371/journal.pone.0071177 (PMC3743895; doi:10.1371/journal.pone.0071177)
Supplement: Figure S1 — Deacidification mechanisms in cancer cells. Each rectangle bar represents a transporter, enzyme or pump family. The red colored rectangles are up-regulated in our study and the green show down-regulation. Dashed arrows indicate CO2 diffusion across the membrane. (PDF) [file pone.0071177.s001.pdf]

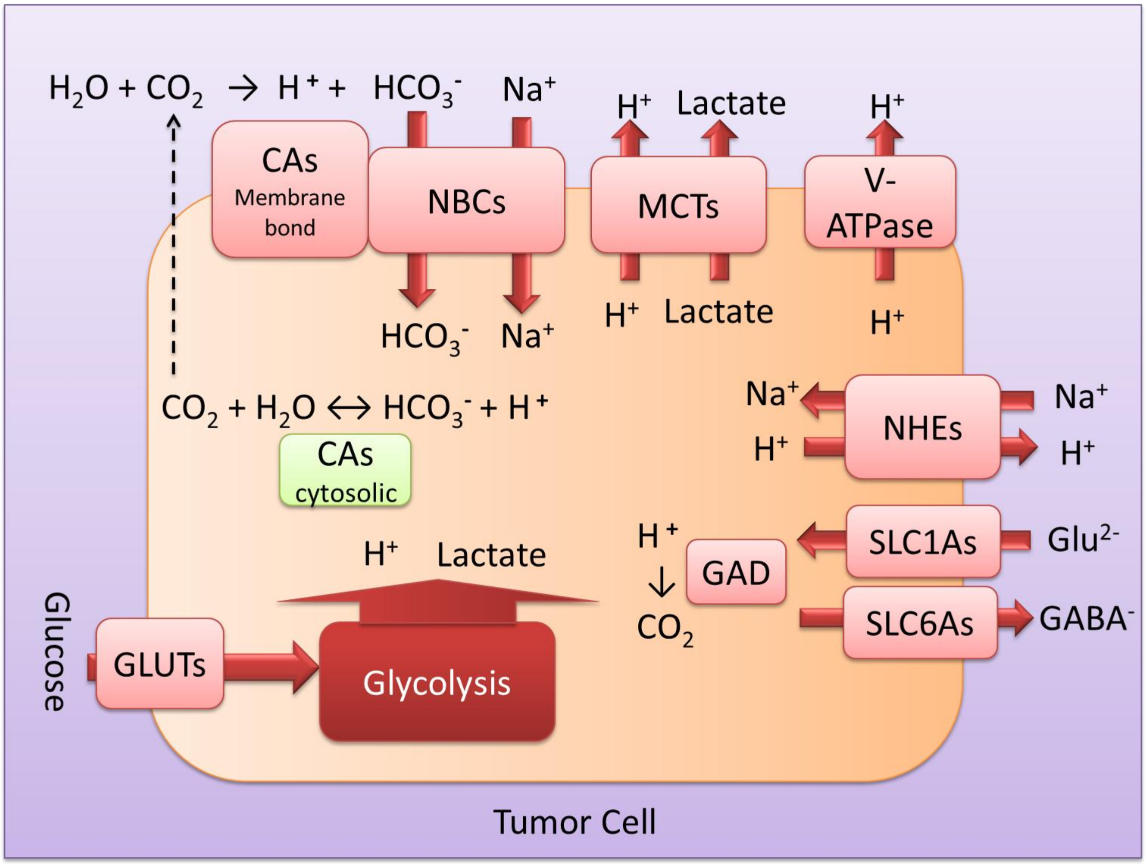

Figure S1: Deacidification mechanisms in cancer cells. Each rectangle bar represents a transporter, enzyme or pump family. The red colored rectangles are up-regulated in our study and the green show down-regulation. Dashed arrows indicate CO_2 diffusion across the membrane.
